# Global Ex-Situ Crop Diversity Conservation and the Svalbard Global Seed Vault: Assessing the Current Status

**DOI:** 10.1371/journal.pone.0064146

**Published:** 2013-05-09

**Authors:** Ola T. Westengen, Simon Jeppson, Luigi Guarino

**Affiliations:** 1 Nordic Genetic Resource Center (NordGen), Alnarp, Sweden; 2 Centre for Development and the Environment, University of Oslo, Oslo, Norway; 3 The Global Crop Diversity Trust, Bonn, Germany; National Rice Research Center, United States of America

## Abstract

*Ex-situ* conservation of crop diversity is a global concern, and the development of an efficient and sustainable conservation system is a historic priority recognized in international law and policy. We assess the completeness of the safety duplication collection in the Svalbard Global Seed Vault with respect to data on the world's *ex-situ* collections as reported by the Food and Agriculture Organization of the United Nations. Currently, 774,601 samples are deposited at Svalbard by 53 genebanks. We estimate that more than one third of the globally *distinct* accessions of 156 crop genera stored in genebanks as orthodox seeds are conserved in the Seed Vault. The numbers of safety duplicates of *Triticum* (wheat), *Sorghum* (sorghum), *Pennisetum* (pearl millet), *Eleusine* (finger millet), *Cicer* (chickpea) and *Lens* (lentil) exceed 50% of the estimated numbers of distinct accessions in global *ex-situ* collections. The number of accessions conserved globally generally reflects importance for food production, but there are significant gaps in the safety collection at Svalbard in some genera of high importance for food security in tropical countries, such as *Amaranthus* (amaranth), *Chenopodium* (quinoa), *Eragrostis* (teff) and *Abelmoschus* (okra). In the 29 food-crop genera with the largest number of accessions stored globally, an average of 5.5 out of the ten largest collections is already represented in the Seed Vault collection or is covered by existing deposit agreements. The high coverage of ITPGRFA Annex 1 crops and of those crops for which there is a CGIAR mandate in the current Seed Vault collection indicates that existence of international policies and institutions are important determinants for accessions to be safety duplicated at Svalbard. As a back-up site for the global conservation system, the Seed Vault plays not only a practical but also a symbolic role for enhanced integration and cooperation for conservation of crop diversity.

## Introduction

The use of genetic diversity to adapt crops to human needs is as old as the Neolithic revolution [1]. In the 1920s the Russian geneticist and botanist Nicolai Vavilov started systematically collecting and conserving genetic diversity as *a resource* for crop breeding, making *ex-situ* (off-site) conservation part of the agricultural R&D system [2]. Crops producing seeds that can be conserved at low relative humidity and low temperature (orthodox seeds) are now commonly conserved *ex-situ* in genebanks. The two-fold rationale for genebanks is, on the one hand, to conserve diversity that is threatened *in-situ* (in farmers' fields or in the wild) and, on the other hand, to make genetic resources accessible to users [3]. In the 1930s, the barley breeder Harry V. Harlan was among the first to sound the alarm on *genetic erosion* of crop genetic resources [4], and, in 1967, a conference in the UN Food and Agriculture Organization (FAO) initiated what has become the genetic resources movement [5]. In the early 1970s, other hallmark conferences laid out practical action plans for the FAO and the Consultative Group on Agricultural Research (CGIAR) to establish an international network of conservation activities and genebanks [6], [7]. While the initial focus was on establishing a small number of genebanks with a global mandate, the FAO currently reports that there are 1750 genebanks around the world [8]. Precarious funding, in combination with less than perfect collaboration and coordination among genebanks, has called into question the ability of many of these facilities to ensure long-term conservation, and genetic erosion *inside* genebanks has become a major concern [8], [9]. The need for proper safety duplication of the world's unique crop genetic resources is therefore an important international priority [8], [10], [11].

**Figure 1:**
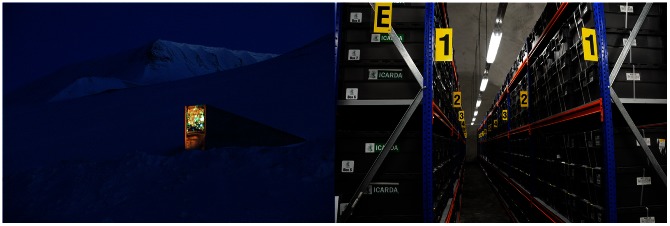
The Svalbard Global Seed Vault. The exterior and interior of the Svalbard Global Seed Vault. Printed under a CC BY license, with permission from Photographer Mari Tefre.

**Figure 2:**
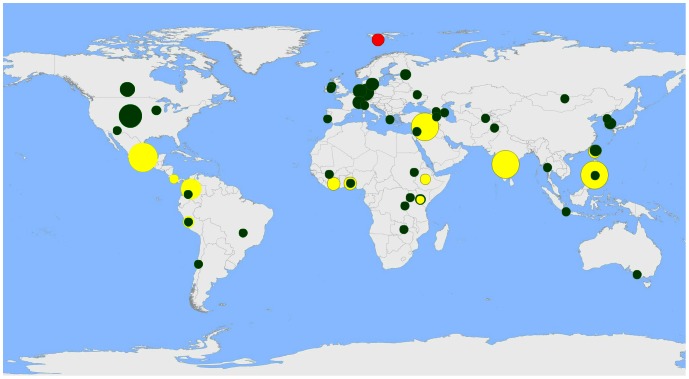
Genebanks with safety deposits in the Svalbard Global Seed Vault. The radius of the circles is relative to the number of samples deposited, and the circle size reflects the size of the deposits according to 25 size classes. Yellow circles are International Agricultural Research Centers, and green circles are regional, national or subnational genebanks. The radius of the red SGSV circle is not relative to the holdings.

The Svalbard Global Seed Vault was established with the “*objective to provide a safety net for the international conservation system of plant genetic resources, and to contribute to the securing of the maximum amount of plant genetic diversity of importance to humanity for the long term in accordance with the latest scientific knowledge and most appropriate techniques*” [12].The Seed Vault is managed in partnership by the Government of Norway, the Nordic Genetic Resource Center (NordGen) and the Global Crop Diversity Trust (the Trust). NordGen is a public regional institute supported by the governments of the Nordic countries, and the Trust an independent international organization based in Bonn, Germany. The Norwegian Ministry of Agriculture and Food is the legally responsible authority for the Seed Vault, and its operation is overseen by an International Advisory Council consisting of international technical and policy experts representing, among others, the FAO, national genebanks, the CGIAR and the Governing Body of the International Treaty on Plant Genetic Resources for Food and Agriculture (ITPGRFA). The Seed Vault provides free-of-charge, long-term storage of duplicates from genebanks around the world and works as an insurance policy against incremental or catastrophic loss of the original collections (Fig. 1). The international community has called for an effective, efficient and sustainable global system to conserve Plant Genetic Resources for Food and Agriculture (PGRFA) in the Global Plan of Action for Plant Genetic Resources for Food and Agriculture (GPA) [13], [14] and the ITPGRFA [15]. The Seed Vault has, in its five years of operation, become a cornerstone in the global system emerging from within this international policy and legal framework, and good progress has been made towards the target of duplicating all the distinct accessions of PGRFA conserved as orthodox seeds around the world. At its fifth anniversary in February 2013, the collection stood at 774,601 seed samples, originating from 95% of the 193 UN member states. All seed samples are safety duplicates of accessions already stored in conventional genebanks, with 53 genebanks having deposited material so far (Fig. 2).

While the idea of establishing an international collection in the permafrost at Svalbard dates back to the early 1980 s, it took almost 20 years before the technical, legal and political context allowed the idea to be realized [16]. The most important factors were the long, and at times politically polarized, international processes and negotiations regarding the conservation and sustainable use of PGRFA [17]. Of particular importance is the legally binding ITPGRFA [15], which, in effect, was a prerequisite for the establishment of the Seed Vault [16], [18]. In exercising their sovereign rights over genetic resources, the Contracting Parties to the ITPGRFA agreed to make their genetic diversity and related information about the crops stored in their genebanks available through a Multilateral System (MLS) for Access and Benefit Sharing (ABS) [15]. It is in this spirit of global collaboration to conserve a resource for which all countries are interdependent that the Seed Vault was established. The management of the Seed Vault has, in its first five years, prioritized storage of material conserved under the terms of the Treaty. But while the Treaty is the international legal and policy framework, the Seed Vault is not a direct instrument of the Treaty. Since the Seed Vault's purpose is to secure as much as possible of all the distinct PGRFA in the world, political and property-rights issues are addressed by ensuring that there is no transfer of legal ownership by depositing seeds in the Seed Vault in its Standard Deposit Agreement (SDA) [19]. Accessions can only be returned to the genebank that deposited them, and distribution to other users is entirely the responsibility of that genebank. This is a standard procedure for safety duplication of PGRFA [11], [20], and the Seed Vault is no exception.

Genebank operation can be grouped into three service categories: genetic resource conservation, distribution, and information assembly and management [21]. To properly fulfill the conservation function, the accessions of genetic resources must be safety duplicated. The new edition of FAO's Genebank Standards, which are still in draft form at the time of writing, devotes a section to guidelines for safety duplication [11]. Because of the costs involved, developing a rational approach to safety duplication is a priority in international collaboration in global *ex-situ* conservation efforts [20]. The new FAO draft standards state that “*a safety duplicate sample for every original accession is stored in a geographically distant area, under the same or better conditions than those in the original genebank*” and, furthermore, that “*the location is chosen to minimize possible risks and provides the best possible storage facilities. To minimize risks that can arise in any individual country safety duplication will be ideally undertaken outside that country.*” [11]. The draft Genebank Standards explicitly state that Svalbard Global Seed Vault now offers a safety-duplication facility which complies with its recommendations.

This is in line with conclusions reached in 2004 by the international committee that undertook the feasibility study for establishing the Seed Vault. That committee formulated the need for the Seed Vault based on the following threats to the *ex-situ* system: “*Individual genebanks are vulnerable to a host of problems that can endanger their collections, including poor management, lack of adequate funding, equipment failures, and natural catastrophes, civil strife, war, and acts of terrorism.*” [22]. The committee concluded that Svalbard is a unique and appropriate location for an international safety duplication facility due to several security, physical and political factors: 1) The permafrost offers natural freezing and provides unparalleled insulation properties for the seeds, even in the unlikely case of breakdown in the cooling equipment; 2) Svalbard offers a unique combination of remoteness and accessibility, providing security from human-related dangers while at the same time allowing transportation of seeds in and out; 3) Military activity is prohibited under the terms of the International Treaty of Svalbard; 4) The political situation is stable, and the highly competent local government is supportive; 5) The location inside a mountain increases security, and the area is geologically stable with low radiation levels; and 6) Norway has the necessary trust in the international efforts on PGRFA conservation [22]. On the basis of this feasibility study the Norwegian government decided to put into practice these plans; in June 2006, the five Nordic prime ministers participated in the cornerstone-laying ceremony at Svalbard, and in February 2008 the Seed Vault opened officially.

In this paper, we assess the completeness of the Seed Vault collection in relation to global conservation efforts and discuss the role of the Seed Vault as back-up storage site in a global *ex-situ* conservation system for PGRFA. We assess available data on the world's collections of PGRFA and compare these results to statistics on what is currently backed-up in the Seed Vault. We establish five different benchmarks for the assessment: 1) The total number of collections and accessions reported in the FAO database World Information and Early Warning System on PGRFA (WIEWS) [23]; 2) the number of *distinct* accessions, as estimated in the Second Report on the State of the World's Plant Genetic Resources for Food and Agriculture (SoW2) from the FAO [8]; 3) the total number of accessions in the GENESYS accession level database [24]; 4) the number of accessions covered by the ITPGRFA, and; 5) the number of accessions held in the ten institutions with the largest number of accessions for a selected array of important food crops. Based on the different framings of the global *ex-situ* collections represented by these five benchmarks, we discuss crop and institutional coverage of the current safety duplication collection in the Seed Vault.

## Methods

We analyzed the current status of the Svalbard Global Seed Vault collection in relation to two different databases on global holdings of PGRFA: 1) the genebank-level data in the World Information and Early Warning System on PGRFA (WIEWS) [23] maintained by the FAO and 2) the accession-level data in GENESYS [24]. As of February 2013, data in the WIEWS database covered more than 190 countries and 1750 genebanks, and GENESYS covered 2,348,549 accessions from 365 participating genebanks in Europe, the USA and the international CGIAR centers. Database downloads of the WIEWS and the GENESYS databases were provided by database managers at the FAO and Bioversity International, respectively. The Svalbard Global Seed Vault database is managed by NordGen, and we also used a download of the accession-level descriptors from that database. Although all these datasets are freely available on the internet, it is not possible to carry out the analyses we required online. We used a PostgreSQL [25] database with data from all three databases (updated as of 01.01.2013) to make taxonomic and institutional queries.

We first categorized the WIEWS and GENESYS data taxonomically and selected relevant genera for our analysis. We included only genera represented with more than 1000 accessions conserved *ex-situ* globally according to WIEWS and those with taxa suitable for *ex-situ* conservation as seeds, following Kew's Seed Information Database (SID) v.7.1 [26] to classify genera according to the predominance of recalcitrant, intermediate or orthodox seed types. We eliminated those that are not currently represented in the Seed Vault, thereby assuming that the genera in the current Seed Vault collection are representative of the genera suitable for conservation of this type. We further classified genera into crop groups (e.g., Cereals, Food Legumes, etc.) by using the classification scheme from FAO's SoW2 [8]. We estimated the number of distinct accessions by using the same method as FAO in SoW2 and counted: a) Accessions in national collections with origins reported from the country of the genebank; b) The international collections held in trust for the world community by the International Agricultural Research Centers (IARCs).

Furthermore, we assessed the number of accessions covered by the global legal framework of the ITPGRFA. We estimated the number of accessions that are covered by the Treaty by extracting data on all taxa covered in the Treaty's Annex 1 from all institutions located in countries that are parties to the Treaty. In addition, we included the collections which are part of the multilateral system under Article 15 agreements with IARCs. Information on participation in the Treaty and the Article 15 collections is available from the ITPGRFA website [27]. We excluded the IARCs with Article 15 agreements in the first query to avoid counting them twice. To determine which taxa to include, we used the Annex 1 list elaborated for an analysis of the taxonomic composition of the collections in the European genebank network [28]. Because of exceptions excluding and including certain species and genera within some crops in the Annex 1 crop list, the Treaty analysis was conducted at the species level for the collections not covered by Article 15.

To assess institutional coverage in the Seed Vault's holdings, we identified the world's ten largest collections (according to WIEWS) for a selection of important food crops. We compared this list of institutions and the number of accessions they hold with the current representation of these collections in the Seed Vault.

## Results and Discussion

On the fifth anniversary of the Svalbard Global Seed Vault on February 26, 2013, the total safety back-up collection stood at 774,601 seed samples deposited by 53 institutions from around the world (Fig. 2). Twelve of these are international, and the rest are regional, national, or subnational, including national agricultural research systems, universities and NGOs. The total number of accessions in the recent FAO WIEWS database is 7,205,007.

From the list of genera with more than 1000 accessions stored in genebanks, we removed names that did not refer to actual genera, combined synonymous genera when warranted, and removed six non-PGRFA genera: *Arabidopsis, Picea, Pinus, Populus, Pseudotsuga* and *Rhododendron*. This resulted in a list of 206 genera. Based on the assumption that the current Seed Vault collection is representative in terms of taxa currently commonly stored *ex-situ* as seeds, we excluded all genera for which no accessions are stored among the current 774,601 in the Seed Vault (*Amygdalis, Anacardium, Ananas, Annona, Bohemeria, Carya, Colocasia, Dioscorea, Ficus, Gladiolus, Ilex, Jathropha, Juglans, Malus, Manihot, Morus, Opuntia, Oxalis, Phoenix, Pistacia, Prunus, Punica, Tulipa, Ullucus, Vitis, Zingiber,*), including genera with recalcitrant and intermediate seeds *(Bactris, Camellia, Carica, Castanea, Cinnamonum, Citrus, Cocos, Coffea, Corylus, Curcuma, Durio, Elaeis, Garcinia, Hevea, Mangifera, Musa, Nephelium, Persea, Piper, Poncirus, Pouteria, Quercus, Salix, Theobroma*), ending up with a final list of 156 genera. The total number of accessions for these 156 genera was 5,979,663 in WIEWS. By using the FAO's method for estimating the number of distinct samples, we identified 2,185,452 distinct accessions in national and IARC collections. And, by using the information on participation in the ITPGRFA and the Annex 1 crop list from [28], we identified 2,625,646 Annex 1 accessions. Together with the 693,752 accessions held by Article 15 institutions according to the Treaty webpage, the total number of accessions covered by the ITPGRFA is about 3.3 million accessions. This ITPGRFA coverage estimate is for the total number of accessions, not only distinct accessions. Our estimates of the size of global *ex-situ* collections of selected genera and crop groups are presented in Table 1, and a list of the 156 crops with more than 1000 accessions in genebanks worldwide is presented in supplementary Table S1.

**Table 1:** Selected crop genera and their status in different databases.

Crop	WIEWS total	GENESYS total	WIEWS distinct	WIEWS ITPGRFA	SGSV (total)
*Triticum* (Wheat)	855,639	367,994	270,237	599,876	145,698
*Oryza* (Rice)	773,948	192,983	440,313	553,235	145,540
*Hordeum* (Barley)	469,590	171,603	138,722	339,448	61,390
*Zea* (Maize)	323,802	104,518	134,185	145,921	32,822
*Sorghum* (Sorghum)	235,690	88,801	75,355	167,769	40,695
*Avena* (Oat)	131,332	56,489	24,619	79,349	11,302
*Pennisetum* (Pearl millet)	65,447	24,910	37,024	89,688	20,444
*Setaria* (Foxtail millet)	46,606	3,193	5,623	1,675*	2,519
*Aegilops* (Goatgrass)	42,268	16,238	19,453	5,173*	3,525
*Triticale* (Wheat x Rye)	40,470	2,833	27,587	31,042	21,455
*Eleusine* (Finger millet)	35,382	7,516	14,602	38,105	7,636
*Amaranthus* (Amaranth)	28,313	5,620	14,290	316*	1,300
*Secale* (Rye)	21,452	15,592	6,091	13,633	935
*Chenopodium* (Quinoa)	16,263	1,648	9,028	34*	111
*Eragrostis* (Teff)	8,820	1,760	6,126	152*	38
**Cereals**	**3,095,022**	**1,061,698**	**1,223,255**	**2,065,416**	**495,410**
*Phaseolus* (Common bean)	262,491	96,433	98,890	246,606	35,230
*Glycine* (Soybean)	230,091	54,797	74,890	2,102*	17,778
*Vigna* (Cowpea)	149,590	48,993	69,302	91,731	26,129
*Arachis* (Groundnut)	128,435	28,489	31,191	22,857*	14,462
*Cicer* (Chickpea)	98,319	48,347	48,432	115,802	28,872
*Pisum* (Pea)	95,290	37,258	20,783	69,193	9,670
*Vicia* (Fababean)	81,470	36,343	32,632	75,492	11,573
*Lens* (Lentil)	58,430	19,557	20,589	56,685	11,946
*Cajanus* (Pigeon pea)	40,820	13,707	31,702	53,326	10,076
*Lupinus* (Lupin)	38,053	13,567	7,503	183*	591
*Psophocarpus* (Winged bean)	4,217	454	1,582	88*	12
Food Legumes	1,187,206	397,945	437,496	734,065	166,339
*Medicago* (Alfalfa)	92,019	27,946	28,413	9,408	10,912
*Trifolium* (Clover)	74,707	34,019	24,062	6,057	3,944
*Panicum* (Switchgrass)	48,850	20,448	12,342	2,273*	2,195
**Forage Crops**	**389,225**	**161,796**	**139,298**	**47,995**	**35,103**
*Solanum* (Potato, Eggplant, Tomato)	147,566	58,781	52,180	48,241	15,733
*Ipomoea* (Sweet potato)	35,478	7,927	16,369	22,864	19,36
**Roots and Tubers**	**183,044**	**66,708**	**68,549**	**71,105**	**17,669**
*Brassica* (Cabbage)	101,353	27,889	26,025	63,624	7,121
*Capsicum* (Peppers)	73,520	21,831	23,945	1,163*	2,163
*Lycopersicon* (Tomato old name)	59,039	23,863	10,801	475*	2,866
*Cucumis* (Cantaloupe)	44,402	16,674	8,000	1*	2,428
*Cucurbita* (Squash)	39,599	14,795	14,411	2,612*	780
*Allium* (Onion)	30,064	12,800	9,816	0*	630
**Vegetables**	**397,069**	**137,084**	**107,672**	**73,209**	**17,988**
*Sesamum* (Sesame)	50,462	3,204	17,946	1*	1,727
*Helianthus* (Sunflower)	39,850	10,921	5,575	20,509	1,811
*Carthamus* (Safflower)	29,195	3,494	6,079	0*	511
**Oil Crops**	**140,196**	**21,030**	**39,729**	**20,511**	**4,068**
**Major crops total (taxa with more than 1000 accessions in ** ***ex-situ*** **)**	**5,979,663**	**2,040,606**	**2,185,452**	**3,117,472**	**766,292**
**All crops total (all taxa in WIEWS)**	**7,205,007**	**2,334,747**	**2,498,098**	**3,319,398**	**774,601**
**Collections**	**1,366**	**365**	**na**	**na**	**53**

Column headings: *WIEWS total* reports the total number of accessions in the World Information and Early Warning System of Plant Genetic Resources for Food and Agriculture database; *GENESYS total* reports the total number of accessions in the GENESSYS database; *WIEWS distinct* reports the estimates of the number of distinct accessions in the WIEWS database; *WIEWS ITPGRFA* reports the estimate for accessions covered by the International Treaty on Plant Genetic Resources for Food and Agriculture, including Annex 1 crop representation in genebanks located in Contracting Parties and the collections in international genebanks under Article 15 agreements (Exclusion of species and lumping of genera under common crop names are done according to the Annex 1crop list for collections not under Article 15); SGSV total reports the current total holding in the Seed Vault. The numbers reported for crop groups are for 156 crop genera with more than 1000 accessions in genebanks worldwide according to WIEWS (See table S1 for full list). *Genera not represented in the Annex 1 list of the ITPGRFA.

In our assessment of institutional coverage, we included only food crops and excluded fiber, sugar, oil and forage crop genera (*Aegilops, Carthamus, Dactylis, Festuca, Gossypium, Linum, Medicago, Panicum, Saccharum, Trifolium*) from the list of 39 genera with most accessions in WIEWS, resulting in a list of 29 genera for which we harvested data on the holdings of the ten largest institutional collections (Table 2, Table S2). For these 29 crop genera, on average 3.8 of the ten largest collections of each genus are currently backed-up at Svalbard. Considering the legal and institutional arrangements of all the individual collections among the ten largest holders, we find that, on average, 5.5 are either already represented in the Seed Vault or covered by existing SDAs. Genebanks in 26 countries with the ten largest collections in the 29 crop genera have not yet safety duplicated in the Seed Vault. All of the CGIAR centers among the top-ten holders have duplicated all or part of their collections.

**Table 2:** Representation in the Seed Vault of the world's 10 largest collections of selected crop genera.

Genus (major crops)	IARCs and Countries of location of 10 largest collections	No of collections in SGSV	No of collections under SDA conditions
*Oryza* (Rice)	IRRI (107), India (0), China (0,0,0), Japan (0), South Korea (4),****USA (20), WARDA (57)	4	5
*Triticum* (Wheat)	CIMMYT (69), USA (17), China (0), India (0), ICARDA (95),****Japan (0), Russia (3), Italy (0), Germany (5), Australia (0)	5	7
*Hordeum* (Barley)	Canada (22), USA (23), Brazil (0), ICARDA (97), Japan (0),****Germany (11), China (0), South Korea (19), Russia (6), Ethiopia (0)	6	7
*Sorghum* (Sorghum)	ICRISAT (83), USA (13, 62), China (0), India (0), Ethiopia (0),****Brazil (0), Kenya (10), Japan (0), Australia (0)	4	7
*Zea* (Maize)	CIMMYT (86), Portugal (0), USA (4), China (0),****Mexico (0,0,0,0), Russia (10), India (0)	3	5
*Glycine* (Soybean)	China (0,0,0), USA (45), South Korea (11),****AVRDC (0), Brazil (0,0), Japan (0), Russia (0)	4	6
*Phaseolus* (Common bean)	CIAT (84), USA (5), Brazil (0,0,0), Mexico (0,0),****Germany (19), China (0), Russia (3)	5	6
*Vigna* (Cowpea)	IITA (74), China (0), USA (1), India (0,0,0),****AVRDC (76), Philippines (8), Japan (0), Brazil (0)	4	7
*Avena* (Oat)	Canada (13,0), USA (26), Russia (2), Germany (7),****Kenya (0), Australia (0), China (0), UK (0), Poland (0)	5	7
*Arachis* (Groundnut)	ICRISAT (58), India (0,0,0), USA (3), Argentina (0,0),****Niger (0), China (0,0)	2	3
*Cicer* (Chickpea)	ICRISAT (85), India (0,0), ICARDA (79), Australia (0), USA (0), Iran (0), Pakistan (19),****Russia (3), Turkey (0)	6	7
*Solanum* (Potato,****Eggplant, Tomato)	Russia (3), AVRDC (18), France (0), CIP (59),****USA (98,0,0), Germany (15), India (0), Japan (0)	5	6
*Pennisetum* (Pearl millet)	ICRISAT (88), Brazil (0), India (0,0), France (0),****Canada (0), India (0), Niger (0), Uganda (0), USA (13)	3	4
*Brassica* (Cabbage)	India (0,0,0,0), China (0,0), Australia (0),****Japan (0), Russia (3), UK (0)	2	3
*Vicia* (Fababean)	ICARDA (63), Russia (3), Australia (0), Germany (14), China (0), Italy (0),****Spain (0), USA (11), Turkey (0), Bulgaria (0)	5	5
*Pisum* (Pea)	Australia (4), Russia (1), ICARDA (62), Germany (16), USA (28),****Italy (0), China (0), UK (0,0), India (0)	5	6
*Setaria* (Foxtail millet)	China (0), India (0), France (0,0), Japan (0), ICRISAT (92),****USA (71), Kenya (0), UK (0)	4	5
*Lens* (Lentil)	ICARDA (99), India (0,0), Australia (0), Iran (0),****USA (0), Russia (6), Chile (0), Canada (32), Hungary (0)	5	7
*Cajanus* (Pigeon pea)	ICRISAT (73), India (0,0,0,0), Kenya (4), Philippines (3),****Australia (0), Brazil (0), Nepal (0)	3	7
*Capsicum* (Peppers)	AVRDC (4), USA (5), Mexico (0,0,0), India (0),****Brazil (0,0), Japan (0), Philippines (0)	3	5
*Sesamum* (Sesame)	India (0,0,0), China (0,0), Israel (0), Kenya (0), Brazil (0),****Japan (0), Mexico (0)	1	5
*Eleusine* (Finger millet)	India (0,0,0), ICRISAT (98) Kenya (19), Ethiopia (0),****Uganda (0), Zambia (3), Nepal (0), USA (88)	4	5
*Triticale* (Wheat x Rye)	CIMMYT (114), Russia (0), USA (5), Canada (5), Ukraine (0), Poland (0),****Germany (7), Bulgaria (0), Slovakia (0), Uzbekistan (0)	5	6
*Cucumis* (Cucumbers, True Melons)	USA (23), Japan (0), Russia (0), China (0,0), Brazil (0),****Kazakhstan (0), France (0), Germany (22), India (0)	3	5
*Helianthus* (Sunflower)	Serbia (0), USA (37), China (0), France (0), Brazil (0,0),****Russia (0), Australia (0), India (0), Morocco (0)	2	5
*Lupinus* (Lupin)	Australia (0,0), Germany (6), Russia (1), France (0),****Peru (0,0,0), Spain (0), UK (0)	3	4
*Ipomoea* (Sweet potato)	CIP (30), Japan (0,0,0), USA (0), Papua New Guinea (0),****Brazil (0), China (0), Taiwan ROC (0)	3	3
*Allium* (Onion)	India (0), Russia (0), Japan (0), USA (0,0), Germany (12),****AVRDC (0), UK (0,0), Hungary (0)	3	5
*Amaranthus* (Amaranth)	India (0,0,0), USA (29), Brazil (0), Peru (0), China (0),****Hungary (0), AVRDC (33), Argentina (0)	2	6
Average		3,8	5,5

The table lists the genera with the largest number of accessions conserved *ex-situ*. The list excludes fiber, sugar, oil and forage crop genera (*Gossypium, Medicago, Trifolium, Panicum, Linum, Saccharum, Aegilops, Festuca, Dactylis, Carthamus*). The percentages of the individual collections backed up in the Seed Vault compared to the total number of accessions reported in WIEWS are given in brackets. The collections/countries are sorted by decreasing number of accessions. If a country holds more than one collection the percentage share of all collections are given in the bracket after the first appearance of the country on the list.

### Crop coverage in the Seed Vault collection

Out of the total number of accessions of genetic resources conserved *ex-situ* in the world according to WIEWS, about 10% are currently backed up in the Seed Vault. However, this is an underestimate of the coverage of the current Seed Vault collection with regard to its objective to back-up the *maximum amount of genetic diversity*, which is not the same as the *maximum number of accessions*. It is important to distinguish between numbers and diversity because according to estimates presented in FAO's SoW2 only about 25–30% of the 7.4 million accessions conserved *ex-situ* are distinct, while the remaining are duplicates held within the same institutions or by different ones [8]. The Seed Vault will as far as possible only conserve safety duplicates of *distinct* accessions and avoid internal duplication [19]. Furthermore, the Seed Vault can only back-up accession of taxa suitable for conservation as seeds, whereas WIEWS includes data on a range of taxa not suitable for conservation in this form.

**Figure 3:**
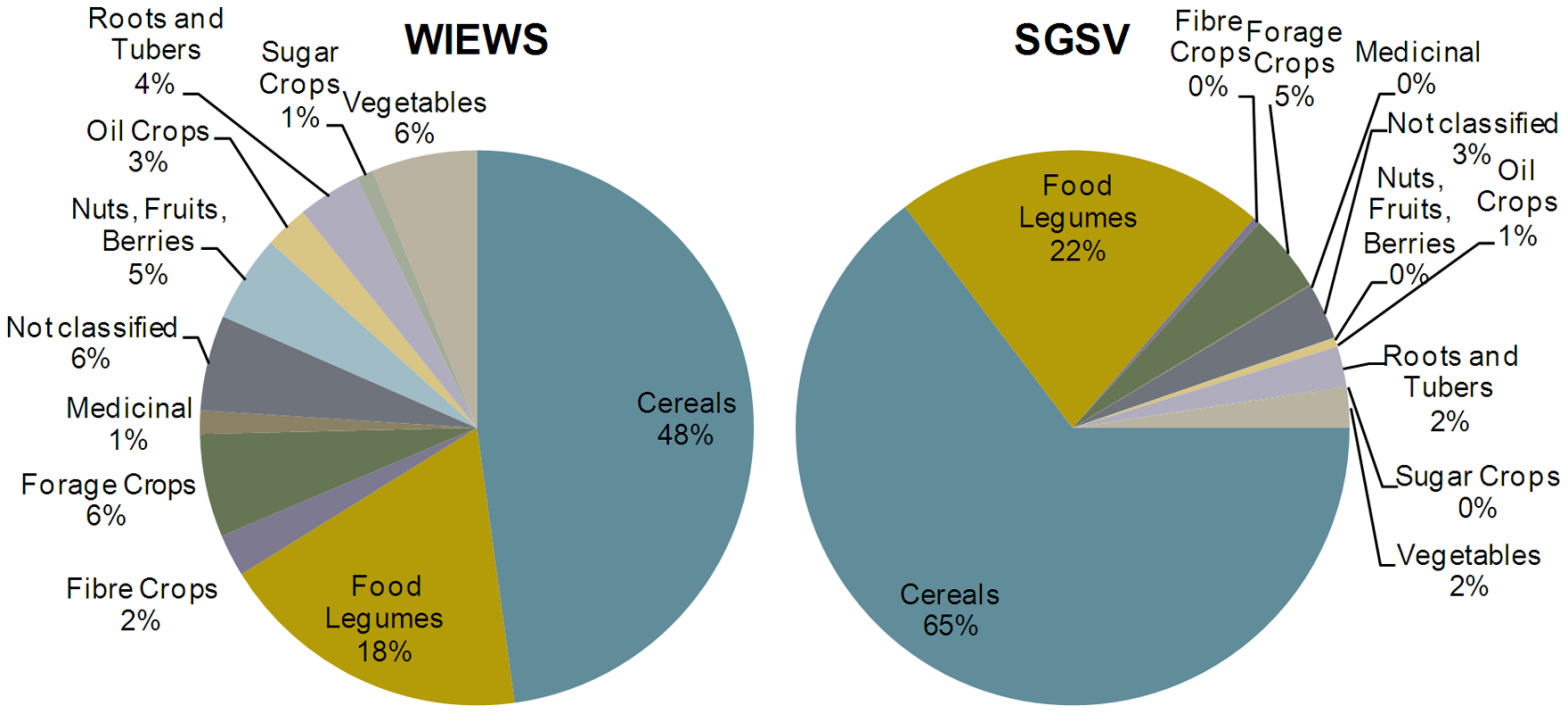
Crop group representation. All genera with more than 1000 accessions reported in the WIEWS database of the FAO and in the safety back-up collection in the Svalbard Global Seed Vault.

We found that the relative representation of crop groups in the current Seed Vault collection is dominated by crop groups containing crop genera with large global production and orthodox seeds. Cereals and food legumes together constitute 87% of the accessions in the Seed Vault, whereas the figure is 65% for the same groups in the WIEWS database (Fig. 3). Among the 206 genera with more than 1000 accessions in WIEWS, 50 are not being stored in the Seed Vault. Twenty-three of these can be explained by having recalcitrant or intermediate seeds, and among the remaining 27 genera not stored at Svalbard are many genera that produce orthodox seeds, but are not normally conserved *ex-situ* because the seeds are not true to type (e.g. *Coffea, Dioscorea, Malus, Manihot, Prunus, Pyrus, Vitis*). In theory, all orthodox seed species can be conserved as seeds, but in practice genebanks predominantly store seeds from crops whose seeds are “true to type”. Most root and tuber crops, for example, are normally asexually (clonally) propagated, as sexual reproduction results in plants that are markedly different from their parents. *Manihot* is an example of a genus with orthodox seeds, but whose propagation is usually done clonally, and, consequently, whose *ex-situ* conservation focuses on *in-vitro*, cryo-conservation or tuber storage in combination with field genebanks. However, there are also examples of tubers where conservation as true seeds is now common, such as potato (*Solanum tuberosum*) [29]. In the case of potato and sweet potato (*Ipomoea batatas*), CIP uses true seed conservation in addition to other methods and that center is currently safety duplicating these seed collections in the Seed Vault [30]. Currently, however, no cassava, taro (*Colocasia*) or yam (*Dioscorea*) accessions are conserved this way in the Seed Vault. We stress that the list of genera in Table 1 and Table S1 is not meant to be comprehensive in terms of PGRFA genera that *can* be stored *ex-situ* as seeds, but rather reflects *the current practice* of most of the world's genebanks.

In terms of total number of accessions, the largest genera in WIEWS are those of wheat (*Triticum*), rice (*Oryza*), barley (*Hordeum*), maize (*Zea*), common bean (*Phaseolus*), sorghum (*Sorghum*), soybean (*Glycine*), cowpea (*Vigna*), potato, tomato and eggplant (*Solanum*), oat (*Avena*), groundnut (*Arachis*), cotton (*Gossypium*) and, cabbage (*Brassica*), all with more than 100,000 accessions stored in genebanks globally. As shown by Kilian and Graner [31], there is a correlation between the aggregate size of *ex-situ* collections of the ten largest crops worldwide and their global acreages in agriculture. We found that the correspondence between number of accessions of crops stored *ex-situ* and agricultural importance goes beyond the big staple crops; comparing the list of species found to “feed the world” in Prescott-Allen and Prescott-Allen (1990) [32] with the WIEWS data showed that only eight of the 103 species providing more than 90% of plant commodities worldwide are from genera represented by less than 1000 accessions conserved in *ex-situ* collections worldwide.

The GENESYS database contains data on 2 million accessions in the 156 genera included in this assessment, corresponding to about one third of the number in the WIEWS database. This proportion also holds for the major crop groups (cereals, food legumes, roots and tubers and vegetables), whereas there is a smaller relative representation of other crop groups, such as oil and fiber crops in GENESYS compared to WIEWS. The GENESYS accession-level database serves a different and broader user-group than does the WIEWS database, most notably breeders and other plant scientists that needs accession-level information. WIEWS does not provide data on descriptors for every accession like genebank databases do, it merely provides data on the number of accessions institutions hold for different taxa. WIEWS is based on a range of sources updated at different times, such as surveys of institutional holdings, reporting on GPA implementation and SoW country reports. GENESYS, on the other hand, taps directly into genebank databases and should therefore be more up to date. The 365 genebanks that have provided data to GENESYS hold a relatively larger share of the 156 genera than we found for the average holding per genebank in WIEWS. A comparative analysis revealed that for some genera there are more accessions in the Seed Vault than the total numbers in GENESYS (e.g., *Eleusine* and *Triticale*) while the median value across the 156 genera is as low as 14% for the same comparison. Not all of the genebanks with safety duplicates in the Seed Vault are data providers to GENESYS, and comparing numbers is not the same as assessing how much the GENESYS providers have duplicated at Svalbard. The reason why we found it interesting to include GENESYS as a benchmark is that GENESYS itself is emerging as the unifying database for the global *ex-situ* conservation system called for in the GPA and the ITPGRFA. Starting with the accession level data from the CGIAR centers, GENESYS now reports if accessions are backed up in the Seed Vault, thereby connecting these two elements of the emerging global system.

The ratio of accessions stored in the Seed Vault vs. the estimated numbers of distinct accessions in WIEWS is about one third. There is good correspondence between the size of the WIEWS total world holdings and the current collection at Svalbard; the ten largest crop genera in terms of distinct accessions are all among the 15 largest genera in the Seed Vault. The cereals *Triticum*, *Sorghum*, *Pennisetum*, *Triticale* and *Eleusine* are represented with numbers corresponding to >50% of the estimate for distinct world holdings. The same is true for *Lens* and *Cicer* among the food legumes. The least well represented cereal genera (in the broad sense used by FAO) are *Amaranthus*, *Chenopodium* and *Eragrostis*, and the least well represented food legume genera are *Lupinus* and *Psophocarpus*. In terms of importance for global agriculture and food security, we found that the genera that include the top four cereals in terms of world production, *Triticum*, *Sorghum*, *Oryza*, and *Zea*, are represented by 54%, 54%, 33% and 24% of the estimates of distinct accessions in WIEWS, respectively. We found a relative under-representation of genera of some crops that are of great importance for food security in parts of the tropics, such as the genera of *Abelmoschus, Amaranthus, Chenopodium, Eragrostis, Ipomoea* and *Lupinus*.

In our assessment of what is distinct in the global conservation system, we have assumed that indigenous accessions are likely to be distinct, while accessions with origins outside the country of the genebank are likely to be duplicates of those stored in the country of origin. We use this method to be consistent with the figures reported in the SoW2. This method is supported by two characteristics of the global system; the international collecting by the IARCs was reduced in the 1990–2000 period and collecting is increasingly done by institutions based in the country where the collecting takes place [8], and, for IARC collecting, it has been a common practice for expeditions to deposit samples in local genebanks. However, this method does not account for accessions collected in the era when PGRFA was largely considered a common heritage, and some international collection missions collected without depositing seeds in local genebanks, in many cases because there were no local genebanks. Neither does the method account for cases where the collected accessions are lost in the local genebanks. Furthermore, it does not account for accessions that are duplicated within a country, which is the case when countries have reported both working collections and base collections in WIEWS, and also in cases where national genebanks are conserving duplicates of IARC accessions. We therefore think that the estimate of distinct accessions is at the higher end of the likely range and consequently that the proportion of distinct accessions conserved in the Seed Vault reported in this paper are conservative. Collection-specific analyses are needed to obtain more accurate estimates of what is distinct in the different collections. Such analyses can to a certain extent be done by comparing passport and descriptor data from different collections [33], but since the necessary information is not always available, expert knowledge on the different collections is often the most useful way to identify the most important collections in terms of unique diversity conserved [34].

Our estimate of the number of genebank accessions covered by the ITPGRFA's Multilateral System is 3.3 million in total and 3.1 million when considering only the 156 genera. Our assessment of representation of genera in the Seed Vault in relation to the ITPGRFA world collection revealed a variety of situations. Some genera are absent or nearly so from the Multilateral System, although they are fairly well represented in the Seed Vault collection (e.g., *Cucumis* and *Linum*). Others are represented by only a small share in the Seed Vault collection compared to the number covered by the ITPGRFA (e.g., *Ipomoea* and *Helianthus*). Since Annex 1 only covers 35 food crops and 29 forages, it represents only a subset of global *ex-situ* conservation holdings, and this is reflected in our assessment. But while the Seed Vault does hold a large number of non-Annex 1 crops, some genera have conspicuously low representation. The genera *Amaranthus, Allium, Capsicum, Chenopodium, Cucurbita, Eragrostis, Glycine, Lupinus* and *Psophocarpus* are examples of important non-Annex 1 food crops with representation in the Seed Vault corresponding to <10% of the distinct accessions in WIEWS. A notable exception to this low representation of non-Annex 1 crops is *Arachis,* where the share of accessions in the Seed Vault vs. distinct accessions in WIEWS is nearly 50%, chiefly because of the global collections maintained by ICRISAT under Article 15.

### Global participation

The four largest national collections in the world are located in the USA, Russia, India and China. The National Plant Germplasm System (NPGS) in the USA and the N.I. Vavilov All-Russian Scientific Research Institute of Plant Industry (VIR) in Russia are both in the process of backing-up their collections at Svalbard, while the National Bureau of Plant Genetic Resources (NBPGR) in India has signed the SDA, but has not yet started safety duplication. The Institute of Crop Germplasm Resources (ICGR-CAAS) in China is not participating so far. Out of the five other national institutions with more than 100,000 accessions in storage, the national genebank in Japan is not currently a depositor, whereas the national genebanks in Brazil, Canada, Germany and the Republic of Korea have all deposited seeds at Svalbard. The most significant origin country gap is India, which appears 40 times in Table 2. However, safety duplication of the Indian collections is expected in the future since most Indian collections in Table 2 are covered by an SDA between the NBPGR and the Seed Vault. In Brazil, USA and Australia, the base collections have safety duplicated at Svalbard while working collections located in other parts of the same countries also appear in Table 2. There is therefore considerable duplication in the WIEWS data from these countries, illustrating that the genus-level estimate for distinct accessions in the previous section is conservative.

Considering the institutional coverage at the genus level, we found that eight genera have SDA coverage for seven of the ten largest global holders. All genera are represented with accessions from at least one of the largest collections. More than 25% of the aggregate holdings of the ten largest collections of the genera *Cajanus, Cicer, Lens, Oryza*, *Pennisetum, Sorghum, Triticale* and *Triticum* are backed up in the Seed Vault today. For another group of genera, less than 1% is safety duplicated at Svalbard: *Sesamum, Allium, Capsicum, Lupinus* and *Brassica.* These differences in institutional coverage among genera can again to a large extent be explained by the high number of safety duplicates from the CGIAR. The genera with relatively high coverage are those for which a CGIAR center has a global conservation and research mandate, and the genera with relatively low coverage are those for which no such mandate exists. That all of the CGIAR centers on the top tenlist have safety duplicated all or parts of the collections at Svalbard shows that the Seed Vault is now an integral part of the CGIAR's genetic resource management. The CGIAR centers are strategically located in important areas of diversity of their mandate crops, and, for many of the genera in Table 2, the location of the centers and national collection holders match well with areas of high importance of conservation: e.g., IRRI, WARDA and the South Korean national genebank for *Oryza*; ICARDA for *Triticum*, *Hordeum, Cicer*, *Vicia, Pisum* and *Lens*; ICRISAT for *Sorghum*, *Pennisetum, Setaria,* and *Cajanus*; CIMMYT for *Zea*; CIAT for *Phaseolus*; IITA for *Vigna*; CIP for *Solanum*; ICRISAT and the Kenyan national genebank for *Eleusine*; and the USA national genebank for *Helianthus*.

National and subnational genebanks typically conserve a higher number of accessions collected in their local region than from other regions [8] and for some genera in Table 2 certain institutions currently not safety duplicating in the Seed Vault are located in areas of important diversity: e.g., *Oryza* from the Indian and Chinese genebanks; *Sorghum*, *Pennisetum* and *Hordeum* from the Ethiopian genebank; *Zea*, *Phaseolus* and *Capsicum* from the Mexican genebank; *Glycine* from the Chinese genebank; *Cicer* and *Lens* from the Iranian and Indian genebanks; *Pennisetum* from the Nigerian genebank; *Cajanus* from the Indian genebank; and *Amaranthus* from the Indian, Peruvian and Argentinian genebanks. Accessions of these genera and genebanks possibly represent important gaps in the current safety collection at Svalbard, but further analyses are needed to see if they are distinct from those already stored in the Seed Vault.

## Conclusion

The high coverage of ITPGRFA Annex 1 crops and of those crops for which there is a CGIAR mandate in the current Seed Vault collection indicates that international policies and institutions are important determinants for accessions to be safety duplicated at Svalbard. Agriculture in all countries in the world has, at least since the beginning of global trade, been dependent on genetic resources originating elsewhere. While some regions have been richer sources of crops than others [2], [35], [36], there are hardly any regions that today do not grow crops which originated in distant lands. Since crops are subject to the same evolutionary imperative as all other organisms (adapt, move or perish), the long-term success of crop cultivation depends on the availability of genetic diversity. Therefore, at varying levels, all countries are interdependent with regard to access to PGRFA. This interdependence has inspired a sense of global unity with respect to the issue of conserving PGRFA. The need for this has been repeatedly articulated throughout the history of the field, where there have been numerous calls for the creation of a global conservation system [37]. The first mandate to address global crop conservation was given to the International Board for Plant Genetic Resources (now Bioversity International), established in 1974, and in 1983 the important ethical and political aspects of the issue led to the creation of an intergovernmental forum at FAO, the Commission on Genetic Resources for Food and Agriculture (CGRFA) [17]. The CGRFA negotiated and developed the GPA and the ITPGRFA as policy instruments for the development of an efficient and sustainable system of *ex-situ* conservation [14], [15]. The ITPGRFA provides an internationally agreed, partial solution to the politically polarized ABS issue that has arguably resulted in institutional proliferation and duplication of conservation efforts. In principle, the ITPGRFA fosters a global system where each distinct accession only needs to be conserved and made available from one (or at most a small number of) long-term storage site with appropriate safety duplication arrangements. The Global Crop Diversity Trust manages an endowment with the objective of “*providing a permanent source of funds to support the long-term conservation of the ex situ germplasm collections on which the world depends for food security*” [38]. The Svalbard Global Seed vault is one of the recipients of such long-term grants, and, by 2012, the Trust had also financed incoming shipment of 75% of the safety deposits stored at Svalbard [38]. In addition to the Trust, the Seed Vault is also supported by a range of other critical institutions and instruments in the emerging global *ex-situ* conservation system. At its establishment in 2007, the Seed Vault was welcomed by the 172 national members, plus the EU, of the CGRFA [39]. The importance of the Seed Vault has been highlighted in SoW2 [8] and in the second GPA [14], and its role in safety duplication will be highlighted in the new international genebank standards from FAO [11]. Most importantly, 53 of the world's genebanks have confirmed the need and importance of the Seed Vault by depositing a substantial part of their collections at Svalbard within its first five years of operation. Thus, the Seed Vault is today well established within the legal and institutional framework of the international genetic resources movement.

### A symbol for the larger cause

The Seed Vault has gained considerable international media attention and even fame [40]. We believe that media reports, though occasionally inaccurate, have contributed to increased public awareness about the importance of crop diversity. While conservation of genetic resources has been part of the environmental movement since the seminal UN conference on the human environment held in Stockholm in 1972, it has often been overshadowed by other issues. The Seed Vault has contributed towards raising the profile of this issue on the broader environmental and food security agenda. In the words of the late Nobel Laureate, Wangari Maathai: “*The significant public interest in the seed vault project indicates that collectively we are changing the way we think about environmental conservation. We now understand that along with international movements to save endangered species and the rainforests of the world, it is just as important for us to conserve the diversity of the world*'*s crops for future generations.*” The Seed vault is not a panacea for securing the future's food supply, but it is an important element in safely conserving the genetic resources necessary for agricultural development. As the UN Secretary General Ban Ki-moon said on the occasion of his visit in the Seed Vault in 2009: “*Sustainable food production may not begin in this cold arctic environment, but it does begin by conserving crop diversity.*”

In light of the huge media attention directed towards the Seed Vault, it is important to stress that it only makes sense as a part of a global conservation system. The conventional genebanks spread around the world are doing the essential job of conserving, regenerating, multiplying and distributing seeds to those that use them for applied and basic research for agricultural development and increased food security. The Seed Vault is, on the one hand, a high-profile environment and development project and, on the other, a low-tech practical solution increasingly serving a basic global need for the safety duplication of seeds held in conventional genebanks, as documented in our analyses. There are important synergies between these two aspects, and the Seed Vault plays an important symbolic role for enhanced integration and cooperation in the global *ex-situ* conservation efforts.

## Supporting Information

Table S1 **Representation of 156 crop genera in WIEWS with more than 1000 accessions stored** ***ex-situ*** **globally.** Only genera commonly stored as seeds are included.
(XLSX)

Table S2 **The ten largest collections worldwide of the 29 largest food crop genera in WIEWS.** Genebanks are identified by WIEWS coding [23].
(XLSX)
